# Reversible Cerebral Vasoconstriction Syndrome Exacerbation After Calcitonin Gene–Related Peptide Inhibitor Administration

**DOI:** 10.1177/19418744231173832

**Published:** 2023-06-05

**Authors:** Megan Zhao, Eric Kaiser, Brett Cucchiara, Jonah Zuflacht

**Affiliations:** 1 14640 Perelman School of Medicine, University of Pennsylvania, Philadelphia, PA, USA; 2 Department of Neurology, 21798 University of Pennsylvania, Philadelphia, PA, USA

**Keywords:** migraine disorders < headache disorders, stroke and cerebrovascular disease < clinical specialty, neurohospitalist < clinical specialty, neurocritical care < clinical specialty

## Abstract

**Background:** Reversible cerebral vasoconstriction syndrome (RCVS) is a non-inflammatory vasculopathy. While most patients have good clinical outcomes, RCVS can be associated with severe brain injury from ischemic stroke, subarachnoid, and intracerebral hemorrhage. **Purpose:** A number of vasoactive medications have been implicated in RCVS, including triptans, amphetamines, antidepressants, and decongestants. Given the role of CGRP in modulating cerebral vasodilation, the possibility of CGRP inhibitors contributing to RCVS has been raised. **Research Design:** Case report at the University of Pennsylvania. **Study Sample:** Patient at the University of Pennsylvania. **Results:** We report a patient with RCVS in which severe exacerbation resulting in multifocal ischemic stroke occurred following administration of the calcitonin gene–related peptide (CGRP) inhibitor fremanezumab. **Conclusions:** It is unclear whether fremanezumab played a role in this patient's case, but CGRP-inhibitor use should be considered as a potential precipiating factor.

## Introduction

Multiple vasoactive medications have been identified as potentially contributing to RCVS and are identified in up to 70% of patients with the condition.^1^ Several important potential agents, such as triptans and ergotamines, play a major role in headache treatment. Given that headache is a frequent presenting symptoms of RCVS, there is the potential for these agents to be misused as symptomatic treatment if the diagnosis of RCVS is not initially identified.^2^ CGRP inhibitors have been recently introduced as both preventative and abortive therapy for migraine. Given the vasodilatory effects of CGRP, there is the concern that CGRP inhibitor use might trigger or worsen RCVS. We present a case of RCVS with marked exacerbation resulting in multifocal ischemic stroke following exposure to fremanezumab, a CGRP inhibitor.

## Case Description

A 55-year-old woman was transferred to our hospital with multifocal ischemic strokes in the setting of intractable headaches over the preceding weeks. Her past history was notable for episodic migraine without aura occurring about once per year and responsive to triptan therapy, mild depression treated with escitalopram, and a bicuspid aortic valve with bioprosthetic valve replacement 2 years prior for which she was taking aspirin. 5 weeks before presentation, she developed sinus congestion and began taking an over-the-counter pseudoephedrine-guaifenesin compound twice daily. 2 weeks later, she discontinued this medication but began experiencing severe constant headache different from her typical migraines, with associated nausea, vomiting, and vertigo. Given her prior response to triptans, she began taking rizatriptan 10 mg up to twice daily without relief.

1 week later, she experienced a sudden-onset severe headache which prompted presentation to a local emergency room. Head CT and brain MRI were negative for any acute process. She was treated with IV fluids, ketorolac, metoclopramide, and diphenhydramine with symptomatic relief and ultimately discharged home. However, her headaches returned, and she re-presented to the emergency room 2 days later. She was again treated symptomatically and discharged. 3 days later, she was evaluated by her outpatient neurologist who initiated treatment with fremanezumab, a CGRP inhibitor, for presumed refractory migraines. She continued to take rizatriptan daily. 6 days later she returned to the emergency room with refractory headaches. As treatment for her headache, she was given oral prednisone 5 mg twice daily with a rapid taper over 1 week and discharged home.

**Figure 1.**
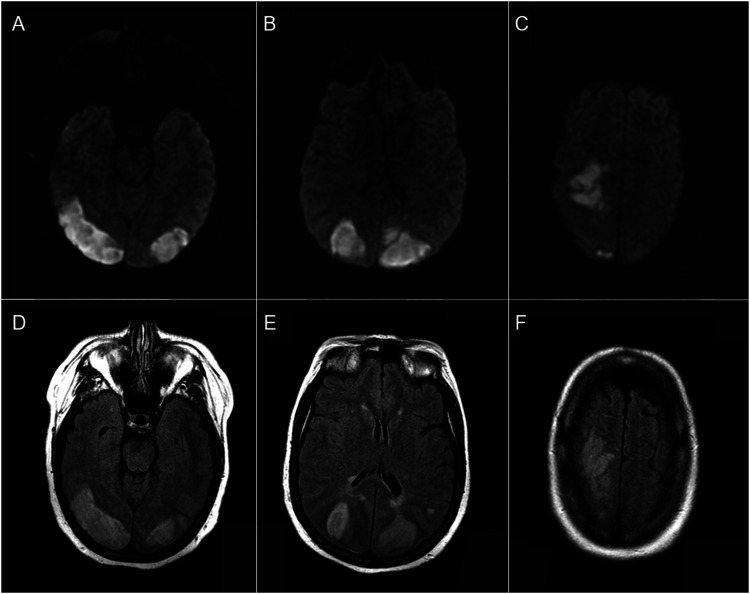
MRI showing multiple, acute bilateral temporal-occipital and right frontal acute infarctions.

**Figure 2.**
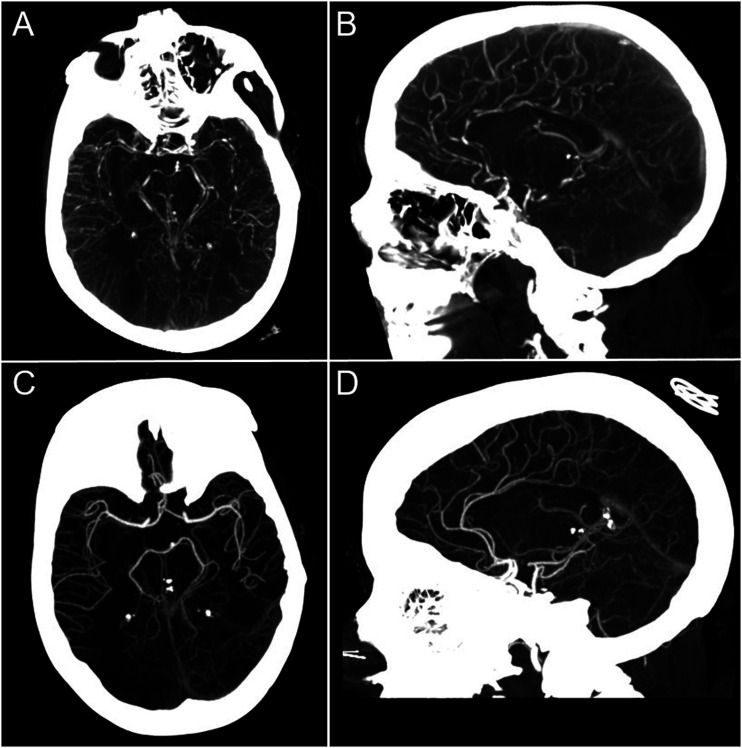
CTA showing multiple areas of segmental stenosis in the anterior and posterior circulations (A and B), near-complete resolution of the multifocal segmental stenoses seen on the original study (C and D).

2 days after discharge and 8 days after starting fremanezumab, she developed left-sided neglect, left hemiparesis, and bilateral visual field impairment. She was admitted to a local hospital where brain MRI demonstrated acute bilateral temporal-occipital and right frontal infarctions (Figure 1). CT angiography (CTA) showed multiple areas of segmental stenosis in the anterior and posterior circulations (Figure 2). She was given a presumptive diagnosis of primary angiitis of the central nervous system (PACNS) and started on high-dose intravenous methlprednisolone. The patient was then transferred to our center for further management. On arrival, neurological examination was notable for a complete left and partial right homonymous hemianopia with macular sparing, left-sided neglect, and left hemiparesis. The National Institute of Health Stroke Scale score was 12. Based on the history and imaging, she was diagnosed with reversible cerebral vasoconstriction syndrome (RCVS). Steroids were discontinued, all vasoactive medications stopped, and she was started on verapamil. She was subsequently discharged to an inpatient rehabilitation unit. At follow-up 3 months later, her strength was significantly improved, but there remained a dense left homonymous hemianopia. Repeat CTA showed near-complete resolution of the multifocal segmental stenoses seen on the original study (Figure 2, C and D).

## Discussion

Reversible cerebral vasoconstriction syndrome (RCVS) is a non-inflammatory vasculopathy characterized by multifocal arterial narrowing of the intracranial circulation. Mechanistically, it is postulated that RCVS results from sympathetic over-activity and dysregulation of cerebral arterial tone.^3^ Thunderclap headache without evidence of subarachnoid hemorrhage and/or multiple recurrent thunderclap headaches are highly suggestive of the diagnosis of RCVS and should prompt a high suspicion for the diagnosis.^4^

Precipitating factors-particularly vasoactive drugs-are identified in up to 70% of patients.^1^ The most important triggers include antimigraine agents such as triptans and ergotamines, decongestant medications such as pseudoephedrine, diet pills and energy-enhancing agents with amphetamine derivatives such as ephedrine, antidepressants like SSRIs and SNRIs, and illicit drugs such as cocaine and amphetamine.^2^ As the name suggests, RCVS is a reversible condition; it is important to identify and subsequently avoid potential triggers which is challenging in patients who chronically suffer from migraines.

In this case, multiple potentially vasoactive medications were used. Our patient was on an SSRI at baseline and used pseudoephedrine for 2 weeks prior to the onset of her headaches. Once the headaches began, she self-treated with multiple doses of a triptan. As triptans are selective 5-HT receptor agonists, stimulation of 5-HT receptors, especially 5-HT_1B_, on blood vessel smooth muscles can lead to cranial vasoconstriction. Given persistent headache, fremanezumab was subsequently administered. Fremanezumab has a robust onset of clinical effect by 7 days post treatment,^5^ which coincides with pharmacokinetic data showing that fremanezumab reaches peak plasma concentrations at 5 days post-administration.^6^ This patient developed multifocal cerebral infarctions 8 days after receiving her first dose, such that an effect of CGRP inhibition in exacerbating her RCVS is temporally plausible.

There has been at least 1 case of RCVS in association with the CGRP inhibitor erenumab.^7^ In that case, a direct cause and effect relationship was suggested by the temporal relationship between exposure to erenumab and the onset of the RCVS. Mechanistically, CGRP is a neuropeptide that plays a critical role as a vasodilator, facilitating increased blood flow in times of cardio or cerebrovascular stress. In cerebral arteries, the trigemino-vascular reflex is activated during vasoconstriction with release of CGRP to counter-balance the vasospasm.^8^ CGRP causes vasodilation by 2 independent pathway. CGRP can bind to CGRP receptors on the endothelium thereby activating cAMP pathways and leading to NO release. Further, in mice models, it has been shown that small molecule CGRP inhibitors worsen cerebral ischemia via collateral dysfunction with diminished collateral flow and reduced reperfusion success.^9^ Therefore, it is plausible that CGRP inhibition could lead to pathological vasoconstriction and the development or exacerbation of RCVS.

Our patient was also given a short course of low-dose oral steroids for refractory headache prior to her worsening. A potential relationship between steroid administration and RCVS exacerbation has been reported in 1 single-center case series.^10^ Whether this is a true effect of steroids remains controversial given the lack of a definitive biological mechanism mediating this effect and the likelihood of confounding by indication, in that steroids are often administered to patients with refractory headache, which may itself be a marker for disease severity and/or risk of progression. In this case series, 30% of the patients treated with steroids received this for a non-neurologic indication, raising additional concerns about confounding due to systemic medical illness which might have impacted outcome. Furthermore, the same group reported an earlier cohort study comparing their RCVS cohort, in which 27% of patients received steroids, to that at another institution where 93% received steroids; outcomes were actually modestly better at the site with routine steroid usage, suggesting that a major negative effect of steroids on outcome is improbable.^11^ In our particular case, our patient received only very low-dose steroids prior to worsening (prednisone 5 mg twice daily), which further argues against this being a major contributor to her worsening.

By definition, RCVS is self-limiting and both the headache and vascular imaging abnormalities resolve within 1–3 months.^12^ While most patients with RCVS have good outcomes, approximately 10% have a more malignant course with stroke leading to severe disability or death.^11^ Early diagnosis and avoidance of vasoactive medications likely reduces the risk of progression to more severe vasoconstriction and ischemic injury and is therefore critical in RCVS. In particular, increased caution should be exercised with CGRP inhibitor use in patients with possible RCVS while further data accrues to better define the safety profile of these agents.

The patient agreed to a waiver of informed consent.
